# Discrimination in the United States: Experiences of lesbian, gay, bisexual, transgender, and queer Americans

**DOI:** 10.1111/1475-6773.13229

**Published:** 2019-10-28

**Authors:** Logan S. Casey, Sari L. Reisner, Mary G. Findling, Robert J. Blendon, John M. Benson, Justin M. Sayde, Carolyn Miller

**Affiliations:** ^1^ Department of Health Policy and Management Harvard T.H. Chan School of Public Health Boston Massachusetts; ^2^ Department of Epidemiology Harvard T.H. Chan School of Public Health Boston Massachusetts; ^3^ Research, Evaluation, and Learning Unit Robert Wood Johnson Foundation Princeton New Jersey

**Keywords:** discrimination, gender identity, Lesbian, gay, bisexual, trangender, queer (LGBTQ) health, Racial/ethnic differences in health and health care, sexual orientation, Social determinants of health, Survey research

## Abstract

**Objective:**

To examine reported experiences of discrimination against lesbian, gay, bisexual, transgender, and queer (LGBTQ) adults in the United States, which broadly contribute to poor health outcomes.

**Data Source and Study Design:**

Data came from a national, probability‐based telephone survey of US adults, including 489 LGBTQ adults (282 non‐Hispanic whites and 201 racial/ethnic minorities), conducted January‐April 2017.

**Methods:**

We calculated the percentages of LGBTQ adults reporting experiences of discrimination in health care and several other domains related to their sexual orientation and, for transgender adults, gender identity. We report these results overall, by race/ethnicity, and among transgender adults only. We used multivariable models to estimate adjusted odds of discrimination between racial/ethnic minority and white LGBTQ respondents.

**Principal Findings:**

Experiences of interpersonal discrimination were common for LGBTQ adults, including slurs (57 percent), microaggressions (53 percent), sexual harassment (51 percent), violence (51 percent), and harassment regarding bathroom use (34 percent). More than one in six LGBTQ adults also reported avoiding health care due to anticipated discrimination (18 percent), including 22 percent of transgender adults, while 16 percent of LGBTQ adults reported discrimination in health care encounters. LGBTQ racial/ethnic minorities had statistically significantly higher odds than whites in reporting discrimination based on their LGBTQ identity when applying for jobs, when trying to vote or participate in politics, and interacting with the legal system

**Conclusions:**

Discrimination is widely experienced by LGBTQ adults across health care and other domains, especially among racial/ethnic minorities. Policy and programmatic efforts are needed to reduce these negative experiences and their health impact on sexual and/or gender minority adults, particularly those who experience compounded forms of discrimination.

## 1 INTRODUCTION

Lesbian, gay, bisexual, transgender, and queer (LGBTQ) people in the United States have experienced a long history of discrimination, including criminalization and classifications as mentally ill, attempts to forcibly change LGBTQ people's sexual orientation and/or gender identity, hate crimes and violence, and exclusion from employment, housing, public spaces, and social institutions.^1^, ^2^, ^3^ And yet, despite this history and despite research examining beliefs about discrimination generally and the consequences of experiencing discrimination (discussed below), relatively few national efforts have been made to systematically study LGBTQ people's reported personal experiences of discrimination.^3^, ^4^, ^5^ While such efforts are hindered by the inherent challenge of surveying a small, dispersed, difficult‐to‐define, and internally diverse population,^6^, ^7^, ^8^ it is nonetheless critically important to study experiences of discrimination because of the established impact of discrimination on health and well‐being.

Research demonstrates that experiencing discrimination or harassment has significant and negative consequences for both physical and mental health.^9^, ^10^ This field of research shows that experiences of enacted stigma, discrimination, and/or harassment induce psychological, behavioral, and physiological stress responses in the body and that the impacts of these reactions accumulate over time,^11^ leading to a wide range of negative health outcomes and health‐related behaviors. Even the anticipation of or mental preparation for discrimination, whether discrimination actually occurs (ie, felt stigma), has significantly harmful effects on health.^12^, ^13^, ^14^

While much related research has focused on the effects of racism^10^, ^15^, ^16^ and sexism on health,^16^, ^17^ these same effects have also been observed in the context of discrimination, harassment, and assault against nonrepresentative samples of LGBTQ people.^18^, ^19^, ^20^, ^21^ In some cases, these effects persist even after basic protection policies have been implemented.^22^ Experiencing discrimination persistently leads to negative health effects for LGBTQ people,^23^, ^24^ and it limits their opportunities and access to critical resources in areas such as health care, employment, and public safety.^21^, ^22^ It also leads to avoidance of care, further amplifying these negative health consequences.^14^ For example, transgender people who have experienced discrimination in health care are more likely than those who have not experienced discrimination to subsequently avoid both preventative and urgent health care services, including needed care due to illness or injury.^22^ This leads to worse health outcomes, including higher likelihood of depression and suicidal ideation or attempts.^14^

Further, these negative consequences for health are likely to be compounded for individuals from multiple minority backgrounds, such as LGBTQ racial/ethnic minorities or LGBTQ women.^18^, ^25^, ^26^, ^27^, ^28^, ^29^, ^30^ Transgender people, with their unique health concerns, may also face special health‐related vulnerabilities as a result of discrimination, including social and economic vulnerabilities that increase health risks.^31^, ^32^ These effects are particularly alarming given that LGBTQ people are significantly less likely than non‐LGBTQ people to have health insurance^31^, ^33^ and therefore may have less access to medical care that could mitigate the adverse health consequences of discrimination.

Few surveys have documented LGBTQ people's personal experiences of discrimination using national data and/or across multiple domains of life. The landmark Institute of Medicine report^6^ on LGBT health in 2011 identified the need for research to overcome some of the methodological challenges that arise in studying LGBTQ population health, such as noninclusion of items to assess sexual orientation and/or gender identity in federal surveys, small population size, stigma, discrimination, privacy, and dispersion in sampling, among others.^8^, ^34^, ^35^ Although some progress has been made, large national probability studies of discrimination across multiple domains among LGBTQ adults remain the exception, rather than the rule. Particularly needed are studies that allow comparisons by race/ethnicity within the LGBTQ population.^3^, ^4^, ^5^, ^6^, ^8^ This study attempts to expand on prior telephone polling methods by examining LGBTQ adults' experiences across many areas of life, drawn from a large national sample of US adults.

This study, alongside complementary articles in this issue of *Health Services Research*, brings a public health perspective to the complexity and pervasiveness of discrimination in the United States today. It was conducted as part of a larger survey fielded in 2017 in response to a growing national debate about discrimination in the United States today,^36^ to understand experiences of discrimination against several different groups in America, including blacks, Latinos, Asians, Native Americans, women, and LGBTQ people. This particular study has four main purposes: (a) to examine the prevalence of discrimination, harassment, and violence against LGBTQ adults specifically because of their sexual orientation and, for transgender adults and gender nonconforming adults, their gender identity; (b) to examine such experiences across multiple domains of life raised as areas of concern among experts,^36^ including health care, education, employment, housing, political participation, police, and the criminal justice system, as well as interpersonal areas including slurs, microaggressions, harassment, and violence; (c) to examine variation in experiences of discrimination within LGBTQ adults by race/ethnicity, as prior research illustrates that racial/ethnic minority LGBTQ adults may be at particular risk for experiencing discrimination; and (d) to examine experiences of discrimination and harassment among a subsample of transgender adults (including those who identified as genderqueer or gender nonconforming), who are also at particular risk for experiencing discrimination.

## 2 METHODS

### 2.1 Study design and sample

Data were obtained from a nationally representative, probability‐based telephone (cell and landline) survey of US adults, conducted from January 26 to April 9, 2017. The survey was jointly designed by Harvard TH Chan School of Public Health, the Robert Wood Johnson Foundation, and National Public Radio. SSRS, an independent firm, administered the survey. Because Harvard researchers were not directly involved in data collection and de‐identified datasets were used for analysis, the study was deemed “not human subjects research” by the Harvard TH Chan School of Public Health Office of Human Research Administration.

The full sample included 3453 US adults aged 18 years and older, including nationally representative samples of blacks, Latinos, Asian Americans, Native Americans, whites, men, women, and LGBTQ adults. This paper examines the subsample of 489 LGBTQ adults, including 282 whites and 201 racial/ethnic minorities and an oversample of 86 transgender adults. Screening questions regarding sexual orientation and gender identity were asked at the beginning of the survey, so that LGBTQ respondents could be identified and asked relevant questions (see Appendix S1). For sexual orientation, respondents were classified as LGBQ if they identified as gay or lesbian, bisexual, or another sexual orientation specified by the respondent that was not heterosexual or straight. For gender identity, respondents were classified as transgender if they identified as transgender male, transgender female, genderqueer or gender nonconforming, or another gender identity specified by the respondent that was not male or female.

The completion rate for this survey was 74 percent among respondents who answered initial demographic screening questions, with a 10 percent overall response rate, calculated based on the American Association for Public Opinion Research's (AAPOR) RR3 formula.^37^ Because data from this study were drawn from a probability sample and used the best available sampling and weighting practices in polling methods (eg, 68 percent of interviews were conducted by cell phone, and 32 percent were conducted via landline), they are expected to provide accurate results consistent with surveys with higher response rates.^38^, ^39^ Surveying LGBTQ populations faces major challenges in constructing adequate sampling frames and sample sizes, as well as a stigmatized respondent population, underreporting, and variations in question wording on sexual orientation and/or gender identity.^6^, ^7^, ^8^, ^34^, ^35^ While federal benchmark data are limited, respondents for this survey were similar demographically to LGB adults in other national, population‐based samples obtaining higher response rates (General Social Survey and National Health Interview Survey),^40^ though federal surveys are also subject to the limitations noted above. We expect these results to be generalizable to the US adult population within a margin of error of ±6.6 percentage points at the 95% confidence interval, while noting the potential for underreporting among the US adult LGBTQ population. See Benson, Ben‐Porath, and Casey (2019) for a further description of the survey methodology.^41^

### 2.2 Survey instrument

In this poll, we analyzed 25 questions about lifetime experiences of discrimination, including adults' personal experiences of discrimination and perceptions of discrimination in the nation. The objective of this study was to examine the extent of discrimination experienced by LGBTQ adults in America, building on question modules in this field adapted from prior surveys on racial and LGBTQ discrimination.^3^, ^4^, ^5^, ^42^, ^43^ We conceptualized discrimination as differential or unfair treatment of individuals based on their LGBTQ identity, and we include discrimination that is “institutional” (based in laws, policies, institutions, and related behavior of individuals who work in or control these laws, policies, or institutions) and “interpersonal” (based in individuals' beliefs, words, and behavior).^8^, ^43^, ^44^,^a^

For this study, we analyzed questions about personal experiences, covering six institutional and seven interpersonal areas of discrimination (full questions and wording in Appendix S1). Institutional areas included employment, education, health care, housing, political participation, and police and courts. Interpersonal areas included anti‐LGBTQ slurs, microaggressions, other people's fear of LGBTQ adults, sexual harassment, being threatened or nonsexually harassed, being harassed or questioned regarding bathroom use, and experiencing violence, among other experiences. We also examined two areas where individuals might avoid seeking help or services due to anticipation or fear of being discriminated against: seeking medical care or the services of police or other authority figures. We examined these numerous domains in order to capture a wide range of possible discriminatory experiences across adults' lives.

Questions were only asked among a random half‐sample of respondents to maximize the number of questions while limiting respondent burden (half‐sample A = 259, half‐sample B = 230). Questions were only asked of relevant subgroups (eg, college‐related questions only asked among adults who had ever applied to or attended college). Questions about harassment (sexual and nonsexual), violence, and avoiding institutions for fear of discrimination were asked about yourself or friends or family members who are also LGBTQ, because of the sensitive nature of the questions and prior literature demonstrating that vicariously experiencing stress (eg, through discrimination experienced by family members) can directly and adversely affect individuals.^45^

### 2.3 Statistical analyses

We first calculated the prevalence of all LGBTQ people who reported they had ever experienced discrimination because of their sexual orientation and/or gender identity in each of the aforementioned domains. Second, we generated bivariate statistics to assess whether experiencing discrimination because of LGBTQ identity was associated with race. Because of the sample size, particularly with split‐sampled questions, responses of nonwhite racial/ethnic minorities were pooled together, and we compared whites to racial/ethnic minorities. Six people were included in overall analyses but excluded from racial/ethnic comparisons because of insufficient race/ethnicity data. Using pairwise *t* tests of differences in proportions, we made uncontrolled comparisons of the weighted percentage of adults reporting discrimination between racial/ethnic minority and white adults, to examine where race/ethnicity affects LGBTQ adults' experiences of discrimination, irrespective of cause. For all analyses, statistical significance was determined at *P* < .05.

We then conducted logistic regression models to assess whether identifying as a racial/ethnic minority remained statistically significantly associated with discrimination after controlling for the following covariates and possible confounders: self‐identified gender (male or female, excluding genderqueer or gender nonconforming due to insufficient sample size, n = 28); age in years (18‐29 or 30+); self‐reported household income (<$25 000 or $25 000+); and education (less than college degree or college graduate). We also examined whether each of these sociodemographic variables was significantly associated with experiencing discrimination across domains. Metropolitan status, region, and health insurance status were omitted from these models for parsimony, due to the sample size. Odds ratios (OR) and 95% confidence intervals (95% CI) were estimated.

Finally, we conducted a subgroup descriptive analysis of transgender adults (n = 86), to assess their experiences separately from the larger LGBTQ population, given that we expected transgender experiences to be unique.^3^ We did not directly compare transgender adults to LGBQ adults because the groups are not mutually exclusive. Due to randomly assigned split sampling of the survey questionnaire, there were some questions that had too few transgender respondents to report these percentages (half‐sample A = 33, half‐sample B = 55). Results are only reported if n > 50.

To compensate for known biases in telephone surveys (eg, nonresponse bias) and variations in probability of selection within and across households, sample data were weighted by household size and composition, cell phone/landline use, and demographics (gender, age, education, race/ethnicity, and census region) to reflect the true population distribution of adults in the country. Other techniques, including random‐digit dialing, replicate subsamples, and random selection of a respondent within a household, were used to ensure that the sample is representative. All analyses were conducted using STATA version 15.0 (StataCorp), and all tests accounted for the variance introduced by weighted data.

## 3 RESULTS

### 3.1 Characteristics of the LGBTQ study sample

Demographic and socioeconomic characteristics of US LGBTQ adults are displayed in Table 1; percentages of LGBTQ adults who have experienced discrimination because of their sexual orientation and/or gender identity are shown in Table 2; adjusted odds ratios of reporting discrimination are shown in Table 3; descriptive analysis of transgender adults is shown in Table 4. All estimates display data weighted using survey weights.

**Table 1:** Characteristics of LGBTQ adults in the study sample (N = 489)^a^

	All LGBTQ adults N = 489	White LGBTQ adults N = 282	Racial/ethnic minority LGBTQ N = 201
	Weighted percentage of respondents^b^		
LGBQ (lesbian, gay, bisexual, and queer)^c^	84	83	84
Cisgender	77	‐	‐
Transgender (including genderqueer and gender nonconforming)^c^	23	25	20
Self‐reported gender			
Male (cisgender and transgender)	38	35	43
Female (cisgender and transgender)	56	58	53
Genderqueer or gender nonconforming	6	6	5
Race			
White (non‐Hispanic)	61	‐	‐
Nonwhite (racial/ethnic minority)^d^	39	‐	‐
Age			
18‐29 y	41	39	45
30 + y	59	61	55
Education			
No college degree^e^	68	62	**77** *
College degree or more	32	38	**23** *
Household income			
<$25 000	36	31	44
$25 000+	55	61	**46** *
Health insurance current status^f^			
Uninsured	15	10	**23** *
Insured, Medicaid	14	16	11
Insured, non‐Medicaid	68	71	65
Area of residence^g^			
Urban	30	26	3
Nonurban	64	67	61
Don't know/refused	6	7	4
US region of residence^h^			
Northeast	23	22	26
Midwest	20	23	17
South	30	33	27
West	20	16	26
Don't know/refused	6	6	4

a Percentage of US LGBTQ population estimated with survey weights to adjust for unequal probability of sampling.

b The sample size shown reflects the total number of respondents in each category. Percentages may not add up to 100% due to rounding and don't know/refused responses that are included in the total n but not reported in Table 1.

c LGBQ and transgender are not mutually exclusive. A person can identify as one or both.

d There were too few LGBTQ‐identified racial/ethnic minority respondents to conduct independent analyses for each racial category (black, Latino, Asian American, Native American), particularly when questions are split‐sampled.

e Including those with some college experience (including business, technical, or vocational school after high school) but no college degree, as well as those with a high school degree or GED certificate or less.

f Primary source of health insurance.

g Nonurban includes suburban and rural.

h Regions defined by US Census Bureau 4‐region definition.

* Different from whites, statistically significant at *P* < .05 (shown in bold).

**Table 2:** Differences between white and racial/ethnic minority LGBTQ adults in reporting discrimination because of their LGBTQ identity^a^

	Subject of discrimination^b^	N	Weighted percent of all LGBTQ adults^c^	Weighted percent of white LGBTQ^c^	Weighted percent of racial/ethnic minority LGBTQ^c^
*Belief in overall discrimination*					
General belief that discrimination against lesbian, gay, and bisexual people exists today in the United States^d^	All LGBTQ adults (total sample)	489	91	92	88
General belief that discrimination against transgender people exists today in the United States^d^	All LGBTQ adults (total sample)	489	91	93	88
*Experiences of institutional discrimination*					
Employment					
Being paid equally or considered for promotions^e^	You (half‐sample A)	245	22	19	28
Applying for jobs^f^	You (half‐sample A)	245	20	13	32*
Education					
Applying to or while attending college^g^	You (half‐sample B)	192	20	20	20
Health care					
Going to a doctor or health clinic	You (half‐sample B)	230	16	20	9
Housing					
Trying to rent a room/apartment or buy a house^h^	You (half‐sample B)	177	22	25	14
Political participation					
Trying to vote or participate in politics	You (half‐sample A)	255	11	7	16
Police and courts					
Interacting with police	You (half‐sample A)	258	16	11	24*
Unfairly stopped or treated by the police^i^	You or LGBTQ friend/family member (half‐sample A)	259	26	26	26
Unfairly treated by the courts^i^	You or LGBTQ friend/family member (half‐sample A)	259	26	23	31
*Experiences of interpersonal discrimination*					
LGBTQ identity‐based microaggressions^j^	You (half‐sample B)	230	53	64	35*
Racial identity‐based microaggressions	You (half‐sample B)	230	18	6	38*
LGBTQ identity‐based slurs^j^	You (half‐sample B)	230	57	65	41*
Racial identity‐based slurs	You (half‐sample B)	230	38	14	53*
People acted afraid because of your LGBTQ identity^j^	You (half‐sample B)	230	15	17	14
People acted afraid because of your race/ethnicity	You (half‐sample B)	230	12	6	23*
Violence^i^	You or LGBTQ friend/family member (half‐sample A)	259	51	57	42
Threatened or nonsexually harassed^i^	You or LGBTQ friend/family member (half‐sample A)	259	57	60	52
Sexual harassment^i^	You or LGBTQ friend/family member (half‐sample A)	259	51	57	43
Harassed while using bathroom^i^	You or LGBTQ family member (half‐sample A)	259	34	32	36
Been told or felt unwelcome because of being LGBTQ^k^	You or LGBTQ family member (total sample)	489	32	34	31
*Actions based on concerns about discrimination*					
Avoided doctor or health care because of concerns of discrimination/poor treatment	You or LGBTQ family member (half‐sample B)	230	18	21	12
Avoided calling the police because of concerns of discrimination	You or LGBTQ family member (half‐sample A)	259	15	11	21
Thought about moving to another area because of personally experienced discrimination^l^	You (total sample)	489	31	31	30

a White and racial/ethnic minority LGBTQ adults aged 18+, excluding n = 6 adults with missing race/ethnicity that are included in the total sample. Most questions only asked among a randomized subsample of half of respondents. Don't know/refused responses included in the total for unadjusted estimates.

b Questions about you are personal experiences only; questions about you or friend/family member ask if items have happened to you or a friend/family member because you or they are part of the LGBTQ community.

c Percent calculated using survey weights. Bolded and starred values show a statistically significant difference between white and nonwhite LGBTQ adults at *P* < .05 using a *t* test.

d Question asked as “Generally speaking, do you believe there is or is not discrimination against [lesbian, gay, and bisexual people OR transgender people] in America today?”

e Equal pay question only asked among respondents who have ever been employed for pay.

f Jobs question only asked among respondents who have ever applied for a job.

g College application/attendance was only asked among respondents who have ever applied for college or attended college for any amount of time.

h Housing question only asked among respondents who have ever tried to rent a room or apartment, or to apply for a mortgage or buy a home.

i Question wording: “Do you believe that you or a friend or family member who is also part of the LGBTQ community has [experienced/been _____] because you or they are part of the LGBTQ community, or not?”

j Question wording: “In your day‐to‐day life, have any of the following things ever happened to you, or not?” and respondent indicated they had experienced this *and* believed this happened because your sexual orientation or gender identity. Slurs = someone referred to you or a group you belong to using a slur or other negative word; Microaggressions = someone made negative assumptions or insensitive or offensive comments about you; People acted afraid = people acted as if they were afraid of you.

k You or a friend/family member who is also part of the LGBTQ community has been told or felt you would be unwelcome in a neighborhood, building, or housing development you were interested in because you are part of the LGBTQ community.

l You have thought about moving to another area because you have experienced discrimination or unequal treatment where you were living.

**Table 3:** Adjusted odds of reporting personal experiences of discrimination across institutional and interpersonal domains among US LGBTQ adults

Institutional discrimination											
N^a^	Employment		Education	Health care	Housing	Political participation	Police and courts			Avoidance	
	Applying for jobs^b^	Equal pay/promotions^c^	College application/ attendance^d^	Doctor or health clinic visits	Trying to rent or buy a house^e^, ^f^	Trying to vote or participate in politics	Interacting with Police	Unfairly stopped or treated by the police	Unfairly treated by the courts	Avoided calling the police due to discrimination concerns	Avoided doctor due to discrimination concerns
	214	213	167	189	151	219	222	221	223	223	193
Race/ethnicity^g^											
White	Ref	Ref	Ref	Ref	Ref	Ref	Ref	Ref	Ref	Ref	Ref
Racial/ethnic minority	**3.16** * (1.35, 7.40)	1.57 (0.66, 3.75)	0.83 (0.24, 2.81)	**0.31** * (0.10, 0.97)	0.31 (0.08, 1.24)	**3.13** * (1.00, 9.73)	1.75 (0.69, 4.48)	0.90 (0.41, 1.96)	**2.80** * (1.26, 6.24)	1.25 (0.50, 3.13)	0.56 (0.19, 1.64)
Self‐identified gender											
Male	Ref	Ref	Ref	Ref	Ref	Ref	Ref	Ref	Ref	Ref	Ref
Female	**0.39** * (0.15, 0.96)	**0.34** * (0.14, 0.82)	2.12 (0.77, 5.86)	1.40 (0.54, 3.66)	0.87 (0.26, 2.96)	**0.28** * (0.09, 0.86)	0.36 (0.13, 0.94)	1.14 (0.50, 2.64)	**0.40** * (0.17, 0.96)	0.89 (0.32, 2.53)	1.11 (0.42, 2.91)
Education											
<College	Ref	Ref	Ref	Ref	Ref	Ref	Ref	Ref	Ref	Ref	Ref
College+	1.12 (0.44, 2.87)	0.92 (0.38, 2.23)	0.46 (0.14, 1.48)	1.26 (0.41, 3.88)	**0.24** * (0.08, 0.74)	1.33 (0.35, 5.09)	1.46 (0.48, 4.45)	2.28 (0.93, 5.57)	1.72 (0.71, 4.17)	1.55 (0.53,4.51)	**3.35** * (1.32, 8.48)
Income											
<25k	Ref	Ref	Ref	Ref	Ref	Ref	Ref	Ref	Ref	Ref	Ref
25k+	0.67 (0.29, 1.56)	0.87 (0.34, 2.23)	1.28 (0.35, 4.76)	0.86 (0.24, 3.09)	0.77 (0.21, 2.86)	0.37 (0.11, 1.22)	**0.31** * (0.10, 0.96)	0.79 (0.32, 1.95)	1.08 (0.46, 2.56)	0.40 (0.14, 1.15)	0.72 (0.25, 2.04)
Age											
18‐29	Ref	Ref	Ref	Ref	‐	Ref	Ref	Ref	Ref	Ref	Ref
30+	0.52 (0.20, 1.33)	1.17 (0.43, 3.24)	2.46 (0.53, 11.39)	2.89 (0.69, 12.12)	‐	0.86 (0.27, 2.72)	**0.35** * (0.13, 0.93)	0.84 (0.36, 1.97)	1.06 (0.45, 2.50)	0.41 (0.15, 1.13)	**0.31** * (0.11, 0.92)

Interpersonal discrimination								
N^a^	LGBTQ identity‐based microaggressions	LGBTQ identity‐based slurs	Sexual harassment	Threats or nonsexual harassment	Violence	Harassed while using the bathroom	Been told/felt unwelcome because you are LGBTQ	Thought about moving to another area
	421	421	222	224	225	225	412	420
OR (95% CI)								
Race/ethnicity^g^								
White	Ref	Ref	Ref	Ref	Ref	Ref	Ref	Ref
Racial/ethnic minority	**0.44** * (0.21, 0.88)	0.51 (0.25, 1.02)	0.72 (0.33, 1.58)	0.79 (0.35, 1.76)	0.52 (0.23, 1.15)	1.43 (0.64, 3.22)	1.07 (0.59, 1.94)	0.95 (0.51, 1.77)
Self‐identified gender								
Male	Ref	Ref	Ref	Ref	Ref	Ref	Ref	Ref
Female	1.11 (0.59, 2.05)	0.97 0.52, 1.82)	0.87 (0.41, 1.86)	0.68 (0.32, 1.45)	**0.40** * (0.19, 0.83)	1.28 (0.59, 2.75)	1.01 (0.57, 1.79)	1.28 (0.70, 2.34)
Education								
<College	Ref	Ref	Ref	Ref	Ref	Ref	Ref	Ref
College+	1.06 (0.56, 2.00)	1.48 (0.79, 2.80)	1.94 (0.87, 4.32)	2.20 (0.96, 5.01)	1.29 (0.57, 2.88)	2.06 (0.89, 4.81)	1.20 (0.66, 2.17)	0.85 (0.45, 1.59)
Income								
<25k	Ref	Ref	Ref	Ref	Ref	Ref	Ref	Ref
25k+	1.46 (0.71, 2.99)	0.92 (0.44, 1.91)	1.04 (0.44, 2.43)	1.16 (0.49, 2.70)	0.82 (0.34, 1.99)	1.43 (0.58, 3.50)	1.22 (0.65, 2.31)	0.97 (0.50, 1.89)
Age								
18‐29	Ref	Ref	Ref	Ref	Ref	Ref	Ref	Ref
30+	**0.44** * (0.21, 0.89)	0.88 (0.41, 1.89)	0.50 (0.22, 1.16)	0.50 (0.21, 1.20)	0.68 (0.29, 1.57)	0.72 (0.31, 1.66)	0.67 (0.36, 1.24)	0.89 (0.46, 1.73)

Abbreviations: CI, confidence interval; OR, odds ratio.

a Individual questions only asked among a randomized half‐sample of respondents. Don't know/refused responses coded as missing.

b Jobs question only asked among respondents who have ever applied for a job.

c Equal pay question only asked among respondents who have ever been employed for pay.

d College application/attendance only asked among respondents who have ever applied for college or attended college for any amount of time.

e Housing question only asked among respondents who have ever tried to rent a room or apartment, or to apply for a mortgage or buy a home.

f Age variable omitted in the housing model due to too few respondents aged 18‐29 who had ever attempted to rent an apartment or buy a house or mortgage.

g White (non‐Hispanic) or racial/ethnic minority (including African American/black, Hispanic/Latino, Asian, American Indian, Alaska Native, Native Hawaiian, Pacific Islander, and other nonwhite identities).

* Significant at *P* < .05 (shown in bold). US LGBTQ adults aged 18+.

**Table 4:** Prevalence of transgender adults reporting discrimination^a^

	Subject of discrimination^b^	N	Weighted percent of transgender adults^c^
*Belief in overall discrimination*			
General belief that discrimination against transgender people exists today in the United States^d^	All transgender adults (total sample)	86	84
*Personal experiences of institutional discrimination*			
Health care			
Going to a doctor or health clinic	You (half‐sample B)	55	10
*Personal experiences of interpersonal discrimination*			
Microaggressions^e^	You (half‐sample B)	55	28
Slurs^e^	You (half‐sample B)	55	38
People acted afraid^e^	You (half‐sample B)	55	18
Been told or felt unwelcome because of being transgender^f^	You or LGBTQ friend/family member (total sample)	86	22
*Actions based on concerns about discrimination*			
Avoided doctor or health care because of concerns of discrimination/poor treatment	You or LGBTQ family member (half‐sample B)	55	22
Thought about moving to another area because of personally experienced discrimination^g^	You (total sample)	86	27

a Transgender adults include transgender, genderqueer, and gender nonconforming adults aged 18+. Most individual questions only asked among a randomized subsample of half of respondents. Don't know/refused responses included in the total for unadjusted estimates.

b Questions about you are personal experiences only; questions about you or LGBTQ friend/family member ask if items have happened to you or a friend/family member because you or they are part of the LGBTQ community.

c Percent calculated using survey weights.

d Question asked as “Generally speaking, do you believe there is or is not discrimination against transgender people in America today?”

e Question wording: “In your day‐to‐day life, have any of the following things ever happened to you, or not?” and respondent indicated they had experienced this *and* believed this happened because your sexual orientation or gender identity. Slurs = someone referred to you or a group you belong to using a slur or other negative word; Microaggressions = someone made negative assumptions or insensitive or offensive comments about you; People acted afraid = people acted as if they were afraid of you.

f You or a friend/family member who is also part of the LGBTQ community has been told or felt you would be unwelcome in a neighborhood, building, or housing development you were interested in because you are part of the LGBTQ community.

g You have thought about moving to another area because you have experienced discrimination or unequal treatment where you were living.

Table 1 shows that a majority of the LGBTQ sample were cisgender (77 percent), with 23 percent identifying as transgender or genderqueer or gender nonconforming. A majority were also white (61 percent), while 39 percent identified as racial and/or ethnic minorities. LGBTQ racial/ethnic minorities were significantly less likely than LGBTQ whites to have a college degree (23 percent vs 38 percent, *P* < .01) and to make $25 000 or more per year (46 percent vs 66 percent, *P* < .04). LGBTQ racial/ethnic minorities were also significantly more likely (23 percent) than LGBTQ whites (10 percent) to be without health insurance (*P* < .02).

### 3.2 Discrimination attributed to sexual orientation and/or gender identity

Table 2 shows the weighted percent of LGBTQ adults, both in aggregate and by race/ethnicity, who reported personally experiencing various forms of discrimination because of their sexual orientation and/or gender identity.^b^ The majority of LGBTQ adults reported personally experiencing interpersonal discrimination: 57 percent said they have experienced slurs and 53 percent said they had experienced microaggressions related to their sexual orientation or gender identity. Similarly, the majority of LGBTQ adults reported interpersonal discrimination either personally or in their immediate friends or family: 57 percent said they or an LGBTQ friend or family member had been threatened or nonsexually harassed because of their LGBTQ identity, and 51 percent said they had experienced sexual harassment or violence because of their sexual orientation and/or gender identity.

More than one‐third (34 percent) of LGBTQ people said that they or an LGBTQ friend or family member has personally been verbally harassed while in a bathroom or been told or asked if they were in the wrong bathroom. Another third (32 percent) said that they or an LGBTQ friend/family member have been told or felt they would be unwelcome in a neighborhood or place to live because they are LGBTQ.

In the context of institutional discrimination, 18 percent of LGBTQ adults reported they have avoided seeking health care for themselves or family members due to anticipated discrimination, while 16 percent reported discrimination in clinical encounters. One‐fifth or more reported personally experiencing discrimination specifically because of their LGBTQ identity across multiple domains of life: when seeking housing (22 percent), equal pay or promotions (22 percent), applying for jobs (20 percent), and applying to or while attending college (20 percent). About one‐quarter of LGBTQ adults said they or LGBTQ friends or family members had also been unfairly treated by the courts (26 percent) or unfairly stopped or treated by police (26 percent) because of their LGBTQ identity.

Importantly, LGBTQ racial/ethnic minorities were more than twice as likely as LGBTQ whites to say they had personally experienced institutional discrimination because of their LGBTQ identity when applying for jobs (32 percent vs 13 percent, *P* < .02) and when interacting with police (24 percent vs 11 percent, *P* < .05). Compared to LGBTQ whites, LGBTQ racial/ethnic minorities reported lower prevalence of some forms of interpersonal discrimination, specifically LGBTQ‐based microaggressions (35 percent vs 64 percent, *P* < .01) and slurs (41 percent vs 65 percent, *P* < .02). However, LGBTQ racial/ethnic minorities had a higher prevalence than whites of reporting race‐based microaggressions (38 percent vs 6 percent, *P* < .01), slurs (53 percent vs 14 percent, *P* < .01), and racial fear (23 percent vs 6 percent, *P* < .01).

### 3.3 Adjusted odds of reporting personal experiences of discrimination in LGBTQ adults

Table 3 reports odds ratios with 95% confidence intervals examining whether race/ethnicity differences in reported experiences of discrimination persist after controlling for pertinent demographic variables, including age, race, gender, education, and income. For institutional discrimination, LGBTQ racial/ethnic minority adults had significantly higher odds than LGBTQ whites for reporting discrimination on the basis of being LGBTQ when applying for jobs, voting or participating in politics, and being treated unfairly by the courts. LGBTQ racial/ethnic minorities had lower odds for reporting LGBTQ‐based discrimination when going to a doctor or health clinic than LGBTQ whites.

Gender also had statistically significant associations in modeling institutional discrimination. Here, LGBTQ females (transgender‐inclusive) had lower odds than LGBTQ males of reporting institutional discrimination when applying for jobs, seeking equal pay or promotions, when trying to vote or participate in politics, and in unfair treatment by the courts. Models did not meaningfully change in sensitivity analyses excluding transgender adults.

Education was also influential: LGBTQ adults with a college degree had significantly higher odds than those without a college degree of reporting they had avoided seeking medical care out of concern they would be discriminated against or treated poorly. LGBTQ adults with a college degree had lower odds of reporting discrimination when seeking housing, compared to those without a college degree.

For interpersonal forms of discrimination, LGBTQ racial/ethnic minorities were less likely than LGBTQ whites to report experiencing LGBTQ‐based microaggressions. LGBTQ adults aged 30 and older also had lower odds of reporting microaggressions, compared to those aged 18‐29. Finally, females were less likely than males to report experiencing LGBTQ‐related violence. No other demographic variables were statistically significant in models of interpersonal discrimination.

### 3.4 Subsample of transgender adults

Table 4 presents the unadjusted percent of transgender adults, where sample size allowed, reporting various experiences of discrimination because of their gender identity and/or sexual orientation. In the context of interpersonal forms of discrimination, 38 percent of transgender adults say they have personally experienced slurs, and 28 percent have experienced microaggressions specifically related to their gender identity and/or sexual orientation. Due to split sampling, there were too few transgender respondents to analyze the question regarding bathroom harassment.

When it comes to health care, 10 percent of transgender people said they have personally experienced discrimination because of their gender identity when going to a doctor or health clinic, and more than one in five (22 percent) said they have avoided seeking health care due to anticipation of discrimination or poor treatment. With regard to the domain of housing, nearly one‐quarter (22 percent) of transgender people reported that they have been told or felt they would be unwelcome in a neighborhood, building, or housing development because they were transgender, while over one‐quarter (27 percent) said they have thought about moving to another area to live because of the discrimination they have already experienced.

## 4 DISCUSSION

In this national US study of reported discrimination among LGBTQ adults, four key findings emerge. First, study results extend prior findings that LGBTQ adults in the United States experience pervasive discrimination across many areas of life.^3^, ^4^, ^5^, ^6^, ^18^, ^19^, ^20^, ^21^, ^24^, ^27^, ^28^ In particular, we found widespread interpersonal manifestations, including slurs, harassment, and violence.

Second, institutional discrimination is also clearly present in health care. Prior research has reported perceived mistreatment in health care settings among LGB and transgender adults.^6^, ^18^, ^32^ In this study, more than one in six LGBTQ adults say they have avoided health care due to anticipated discrimination and experienced discrimination in health care encounters. Among transgender adults, these estimates are even higher. This is particularly worrisome and merits further education and antidiscriminatory policies and training in health care, as avoiding health care can further exacerbate health disparities between LGBTQ and non‐LGBTQ adults.^6^, ^14^, ^22^

Third, LGBTQ racial and ethnic minorities are significantly more likely to report many forms of discrimination, even when controlling for other factors. LGBTQ racial and ethnic minority adults had a significantly lower odds of reporting LGBTQ identity‐based microaggressions relative to whites, though they were more likely than LGBTQ whites to report experiencing racially based microaggressions (not adjusted for demographic characteristics). These results are largely consistent with prior research finding higher reported racial discrimination among racial/ethnic sexual minorities relative to white sexual minorities in public settings, accompanied by both sexual orientation and gender discrimination.^18^ Our findings also support other studies demonstrating that racial/ethnic identity compounds experiences of discrimination in addition to LGBTQ identity in many areas of life.^25^, ^26^, ^27^, ^29^, ^30^

While it is beyond the scope of our results to promote specific policies or practices to end discrimination in the United States, these findings indicate both top‐down (eg, policy) and bottom‐up (eg, community organizations or local initiatives) efforts need to take steps to address this widespread discrimination, on both institutional and (especially) interpersonal levels. For transgender people, housing and health care appear to be major areas of concern, while LGBTQ racial/ethnic minorities face significant obstacles with employment and the legal system. Multisector partnerships are urgently needed to implement interventions, propel policy efforts, and create social change to protect LGBTQ people across different systems, including employment, health care, housing, and legal systems.

In addition, more research is needed that includes both new methods and novel data sources to improve the study of LGBTQ populations, given the current methodological limitations.^6^, ^7^, ^8^, ^34^, ^35^ In particular, research using electronic health record data is a promising approach to further study LGBTQ persons and other small populations, while mobile device or computer apps and other novel methods for data capture may also improve research on the unique experiences of discrimination among LGBTQ persons within the health care system.^8^, ^46^ At a minimum, improving medical and administrative staff training on cultural competency for serving LGBTQ people, as well as improving data collection on sexual orientation and gender identity in health care, is needed.

### 4.1 Limitations

The findings should be viewed with several limitations in mind. First, although we examined a broad range of domains of life, this study covers only a subset of types of discrimination and harassment that LGBTQ people may experience. Second, we asked whether LGBTQ people had experienced these types of discrimination at any point in their life, without regard to timing or severity. This limits the ability to estimate current levels of discrimination and harassment and instead focuses on lifetime experiences.

Third, the prevalence of many sensitive topics, including sexual harassment and violence, is often underreported—particularly on surveys administered by an interviewer,^47^ such as this study—and therefore, the “true” prevalence of LGBTQ people's experiences of discrimination is likely higher than reported herein. Perceptions of various kinds of discrimination (eg, race‐based and sexuality‐based) are also significantly associated with each other,^26^, ^29^, ^30^ and it is not always possible to disentangle these experiences from each other, so asking specifically about LGBTQ‐based discrimination may lead to underreporting of overall discrimination experienced by some respondents. Questions about discrimination based on race/ethnicity and gender (among females only) are examined separately in other articles in this issue.

Fourth, our low response rate is a notable limitation, though evidence suggests that low response rates do not bias results if the survey sample is representative of the study population.^38^, ^39^ Recent research has shown that such surveys, when based on probability samples and weighted using US Census parameters, yield accurate estimates in most cases when compared with both objective measures and higher‐response surveys.^38^, ^39^, ^48^, ^49^ For instance, a recent study showed that across 14 different demographic and personal characteristics, the average difference between government estimates from high‐response rate surveys and a Pew Research Center poll with a response rate similar to this poll was 3 percentage points.^38^ However, it is still possible that some selection bias may remain that is related to the experiences being measured, particularly given the challenges of surveying the LGBTQ population noted earlier.^6^, ^7^, ^8^, ^34^, ^35^, ^36^, ^37^, ^38^, ^39^, ^40^, ^41^, ^42^, ^43^, ^44^, ^45^

Fifth, transgender people are often discriminated against due to their presumed gender or gender identity. Given that trans people may be of any sexual orientation, they may also be discriminated against because of their sexual orientation. Furthermore, some people may not know the difference between sexual orientation and gender identity, so they may discriminate against someone because of their gender but using language about sexual orientation (or vice versa). Therefore, it should be expected that transgender people report experiences of discrimination related to both their gender identity and sexual orientation, and so we report these experiences together, and this study was unable to distinguish between these experiences.

Despite these limitations, this study was strengthened by its probability sampling design and by the breadth of questions asked on LGBTQ‐based discrimination across institutions and interpersonal experiences. It allowed us to examine personal experiences of discrimination and harassment among LGBTQ adults. Our findings may underreport experiences of discrimination and harassment; thus, our results can be considered a lower bound estimate of discrimination and harassment in the United States today. We may also underreport the added burden of discrimination against LGBTQ people who are racial/ethnic minorities.

This study highlights the wide extent to which the LGBTQ adult population as one group experiences discrimination, providing important data to inform national discussions and current policy debates. Yet, future research is needed to assess the distribution and burden of discrimination experiences faced by subgroups within the LGBTQ population.

## 5 CONCLUSION

This study shows that lesbian, gay, bisexual, transgender, and queer adults in America share common, yet diverse experiences of consistent and pervasive discrimination based on their sexual orientation and/or gender identity. Some of the most widespread reported experiences of enacted stigma include slurs, microaggressions, violence, threats, and both sexual and nonsexual harassment. In health care, additional efforts are needed to reduce discrimination against LGBTQ adults. LGBTQ racial/ethnic minorities experience particularly high rates of LGBTQ‐based discrimination in employment and workplace settings and interacting with the legal system, while transgender adults report significant discrimination in both housing and health care. Findings of this study further illustrate the need for substantial changes in institutional policies and practices to protect the civil rights of LGBTQ people. Changes in social norms are also needed to confront stigma and counteract the harmful effects of discrimination in personal interactions. Addressing both institutional and interpersonal discrimination will be vital to improving and ensuring the health and well‐being of LGBTQ Americans.

## ENDNOTES

^a^Institutional and interpersonal forms of discrimination are not mutually exclusive, but this framework is used here for organizational purposes.

^b^There were no statistically significant differences between LGBTQ men and women in their unadjusted reported experiences of anti‐LGBTQ discrimination.
