# Supplementary material for: Discrimination in the United States: Experiences of lesbian, gay, bisexual, transgender, and queer Americans
Source: Health Serv Res. 2019 Oct 28;54(Suppl 2):1454–66. doi: 10.1111/1475-6773.13229 (PMC6864400; doi:10.1111/1475-6773.13229)
Supplement: Supplementary file 2 [file HESR-54-1454-s002.docx]

**APPENDIX S1**

**Survey Questions for “Discrimination in the United States: Experiences of LGBTQ Americans”**

**Sexual orientation/Gender identity screening questions**

**Sexual Orientation Screening Question**

READ TO ALL: We just have a few other questions to help us get to know the people we are talking to. Do you consider yourself to be…? (READ LIST)

Heterosexual or Straight;

Gay or Lesbian;

Bisexual;

Or a different identity I haven’t mentioned (please specify);

(DO NOT READ) Transgender;

(DO NOT READ) Queer;

(DO NOT READ) Refused

(If respondent says something like “I’m just normal.” Do not code under “Other”; please probe for clarity: “I understand, so which of these options do you consider yourself to be…”

(If respondent says “Trans or Transgender” probe with: I understand, we will be asking about your gender identity next. This question is asking about your sexual orientation. Do you consider yourself to be…)

(If respondent says they are both transgender and something else on the list, code both)

**Gender Identity Screening Question asked 1/26/17-2/8/17^[[1]](#footnote-1)^**

I’m required to ask this of everyone. Which of the following do you most identify with? (READ LIST) Male, Female, Transgender male, Transgender female, Genderqueer or gender non-conforming, Or a different identity I haven’t mentioned (please specify); (Do not read) Refused

(IF NECESSARY: IF ASKED ABOUT DEFINITION OF TRANSGENDER: Some people describe themselves as transgender when they experience a different gender identity from their assigned sex at birth. For example, a person who was raised male, but who identifies as a woman might call herself transgender. Some, but not all, transgender people change their physical appearance so that it matches their internal gender identity. Some, but not all, transgender people take hormones or have surgery. A transgender person may be of any sexual orientation – straight, gay, lesbian, or bisexual.)

(IF NECESSARY: IF ASKED ABOUT DEFINITION OF GENDER NON-CONFORMING: Some people think of themselves as genderqueer or gender non-conforming when they do not identify only as a man or only as a woman.)

**Gender Identity Screening Questions asked 2/9/17-4/9/17**

*Asked of everyone unless they indicated they were transgender in the Sexual Orientation Screening Question*. I’m required to ask this of everyone. Some people describe themselves as transgender when they experience a different gender identity from their sex at birth. For example, a person who was raised male, but who identifies as female. Some people who do not identify as either male or female might also call themselves transgender. Do you consider yourself to be transgender?

(DO NOT READ)

Yes, transgender; Yes, genderqueer or gender non-conforming; No; Refused

*If respondent does not identify as transgender or refused the transgender question, then asked:*

What is your gender?

(READ LIST) Male, Female, Or a different identity I haven’t mentioned (please specify); (DO NOT READ) Refused

*If respondent identifies as transgender or genderqueer or gender non-conforming, or not a male or female in the question above:* How would you describe your gender? Select all of the following that apply.

(READ ENTIRE LIST – ENTER ALL THAT APPLY)

Male; Female; Transgender male; Transgender female; Genderqueer or gender non-conforming; Or a different identity I haven’t mentioned (please specify); (DO NOT READ) Refused

*If respondent identifies with more than one identity above:* With which do you identify most?

(READ LIST)

Male; Female; Transgender male; Transgender female; Genderqueer or gender non-conforming; Or a different identity I haven’t mentioned (please specify); (DO NOT READ) Refused

**Discrimination screening questions**

1. Have you ever applied for a job?
2. (Half sample A): Have you ever been employed for pay?
3. (Half sample B): Have you ever applied for college or attended college for any amount of time?
4. (Half sample B): Have you ever tried to rent a room or apartment, or to apply for a mortgage or buy a home?

**General perceptions of discrimination**

Q1. Generally speaking, do you believe there is or is not discrimination against lesbian, gay, and bisexual people in America today? Yes / No / (Volunteered response) Don’t know/Refused

Q2. Generally speaking, do you believe there is or is not discrimination against transgender people in America today? Yes / No / (Volunteered response) Don’t know/Refused

**Institutional Discrimination**

Q3. (Ask if respondent has ever applied for a job): What about you? Do you believe you have ever personally experienced discrimination because you are [insert LGBTQ identity] when applying for jobs? Yes / No / (Volunteered response) Don’t know/Refused

Q4. (Ask if respondent has ever been employed): What about you? Do you believe you have ever personally experienced discrimination because you are [insert LGBTQ identity] when it comes to being paid equally or considered for promotions? Yes / No / (Volunteered response) Don’t know/Refused

Q5. What about you? Do you believe you have ever personally experienced discrimination because you are [insert LGBTQ identity] when interacting with police? Yes / No / (Volunteered response) Have never interacted with police/Don’t know/Refused

Q6. What about you? Do you believe you have ever personally experienced discrimination because you are [insert LGBTQ identity] when trying to vote or participate in politics?

Yes / No / (Volunteered response) Have never tried to vote or participate in politics/Don’t know/Refused

Q7. What about you? Do you believe you have ever personally experienced discrimination because you are [insert LGBTQ identity] when going to a doctor or health clinic? Yes / No / (Volunteered response) Don’t know/Refused

Q8. (Ask if respondent has ever applied to or attended college): What about you? Do you believe you have ever personally experienced discrimination because you are [insert LGBTQ identity] when applying to college or while at college? Yes / No / (Volunteered response) Don’t know/Refused

Q9. (Ask if respondent has ever tried to rent/buy a place to live): What about you? Do you believe you have ever personally experienced discrimination because you are [insert LGBTQ identity] when trying to rent a room or apartment or buy a house? Yes / No / (Volunteered response) Don’t know/Refused

**Interpersonal Discrimination (Against You Only)**

(Rotate items B and C, always ask A last): In your day-to-day life, have any of the following things ever happened to you, or not? How about (INSERT)?

(If respondent asks what “group” means, say: Such as your race, ethnicity, gender (or your sexual orientation or identity).)

Yes, has happened / No, has not happened / (Volunteered response) Don’t know/Refused

Q10. Someone referred to you or a group you belong to using a slur or other negative word

Q11. Someone made negative assumptions or insensitive or offensive comments about you

Q12. People acted as if they were afraid of you

(If Yes to previous question): Do you believe this happened to you because of your race or ethnicity, your gender, (your sexual orientation or gender identity,) or was it for some other reason? You can select multiple answers.

Race or ethnicity, Gender, Sexual orientation, Gender identity, Or some other reason (SPECIFY)

**Interpersonal Discrimination (Against You or Family/Friend)**

(Scramble items a-e; always ask B right after A)

Do you believe that you or a friend or family member who is also part of the LGBTQ community has (INSERT ITEM) because you or they are part of the LGBTQ community? How about (INSERT ITEM)?

(IF NECESSARY: Do you believe that you or a friend or family member who is also part of the LGBTQ community has (INSERT ITEM) because you or they are part of the LGBTQ community?) Yes / No / (Volunteered response) Don’t know/Refused

13. Experienced sexual harassment

14. Been threatened or non-sexually harassed

15. Been unfairly stopped or treated by the police

16. Been unfairly treated by the courts

17. Experienced violence

Q18. Have you or a friend or family member who is also part of the LGBTQ community ever been verbally harassed when entering or while using a bathroom, or been told or asked if you or they were using the wrong bathroom? Yes / No / (Volunteered response) Don’t know/Refused

**AVOIDED DOCTOR**

Q19. Have you ever avoided going to a doctor or seeking health care for you or others in your family out of concern that you would be discriminated against or treated poorly because you or they are part of the LGBTQ community? Yes / No / (Volunteered response) Don’t know/Refused

**AVOIDED POLICE**

Q20. Have you ever avoided calling the police or other authority figures, even when in need, out of concern that you or others in your family would be discriminated against because you or they are part of the LGBTQ community? Yes / No / (Volunteered response) Don’t know/Refused

**FEELING UNWELCOME**

Q21. Have you or a friend or family member who is also part of the LGBTQ community been told or felt as though you wouldn’t be welcome in a neighborhood, building, or housing development you were interested in because you are part of the LGBTQ community? Yes / No / (Volunteered response) Don’t know/Refused

Q22. Have you ever thought about moving or relocating to another area because you experienced discrimination or unequal treatment where you were living? No, I’ve never thought about it / Yes, I’ve thought about moving because of discrimination, but haven’t actually moved / Yes, I’ve actually moved/relocated because of discrimination / (Volunteered response) Don’t know/Refused

1. Note: Phone numbers used for this study were randomly generated from cell phone and landline telephone sample frames, with an overlapping frame design. The sample plan consisted of three basic components: (1) general adult population respondents reached by random digit dialing (RDD) of cell phones or landlines; (2) respondents reached by RDD cell phone or landline, and interviewed only if they were members of one of the racial or ethnic groups at the focus of the study, or if they were members of the LGBTQ community; and (3) pre-screened sample, meaning callbacks to telephone numbers in which respondents who were previously interviewed on the [redacted for review] weekly Omnibus poll indicated they were members of one of these racial or ethnic groups or fell under various definitions of LGBTQ. See [redacted for review] in this issue for full discussion of the survey’s methodology.

   During the first two weeks of this survey’s fielding period, respondents of interest from the pre-screened sample were re-contacted for recruitment into this survey. They were not informed that their previously stated identity was why they were re-contacted. However, some respondents who had previously identified as transgender in the weekly omnibus poll did not identify as transgender in the screening questions for this survey. This pattern took approximately two weeks to observe due to the low incidence of transgender people in the population. It was likely due to the fact that the question asked respondents to select which term they **most** identified with, and some transgender people may be more likely to identify as simply “male” rather than “transgender male,” or “female” rather than “transgender female.” Once the pattern was observed, the gender identity screening question was updated to separate transgender identity from most preferred identification terms. [↑](#footnote-ref-1)
